# Ultrasound‐Assisted Enzymatic Extraction of *Pinus pumila* Nut Protein: Effects on Yield, Physicochemical and Functional Properties

**DOI:** 10.1002/fsn3.70411

**Published:** 2025-06-16

**Authors:** Zhe‐Xuan Mu, Bing‐Xiao Liu, Shuang‐Ding Lai, Zhen‐Yu Wang, Xiao‐Hong Lv, Lin Yang, Hua Zhang

**Affiliations:** ^1^ Department of Special Food and Drug and Biochemical Innovation Research Center, School of Chemistry and Chemical Engineering Harbin Institute of Technology Harbin China; ^2^ Heilongjiang Academy of Forestry Harbin China

**Keywords:** *Pinus pumila* nut protein, plant protein, protein conformational relationship, protein separation technology, response surface methodology

## Abstract

*Pinus pumila* nuts are renowned for their oil, which is abundant in unsaturated fatty acids. Nevertheless, the utilization of their oil meal has been constrained by the dearth of effective deep processing technology. To promote the use of *Pinus pumila* nut protein and environmentally friendly extraction techniques in the food industry, this study used ultrasound‐assisted enzymatic (papain) to extract *Pinus pumila* nut protein (PUEP). The solid–liquid ratio significantly affected the protein yield, and the PUEP yielded up to 28.89%. Particle size, zeta potential, morphology, and color of PUEP are significantly different from traditional protein. The decrease in sulfhydryl content, the increase in the relative content of β‐turns, and the increase in surface hydrophobicity significantly improved the functional properties of PUEP. There were no significant differences in thermal stability, amino acid composition, or molecular weight. PUEP extracted by ultrasound‐assisted enzymatic extraction has a high extraction rate and excellent functional properties. In‐depth analysis of the physico‐chemical and nutritional properties of PUEP can help expand its use in the food industry.

## Graphical abstract

The ultrasound‐assisted enzymatic method (papain) was used to extract Pinus pumila nut protein (PUEP), which increased the yield by more than three times compared to the aqueous method, with an extraction rate of 83.72%.The structure loosening and secondary structure modification resulted in a significant improvement in the functional properties of PUEP. The physicochemical and nutritional properties of PUEP were analysed.

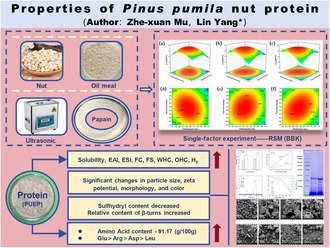

## 1 Introduction

In recent years, there has been an observable increase in public interest in the field of health, precipitated by the significant rise in the incidence of chronic diseases such as malnutrition and immunodeficiency. Tree nuts (e.g., pine nuts, pecans, cashews, hazelnuts etc.) have attracted considerable attention due to their high content of polyunsaturated fatty acids (Karaosmanoglu 2024), high‐quality plant proteins (Derbyshire et al. 2023), polyphenolic compounds, and other biologically active components. *Pinus pumila* (*Pall*.) *Regel* is indigenous to Russia, Korea, Japan, and the Daxinganling region of China (Li et al. 2019), and is utilized for ecological protection (its frigid nature renders it effective in the prevention of soil erosion) and economic exploitation (its seeds are rich in oil and proteins that can be used). The protein content of *Pinus pumila* nut oil meal has been found to be over 30% and has a balanced nutritional value and multifaceted bioactivity. In the context of the mounting worldwide interest in alternative protein sources, pine seed protein has the potential to serve as a supplement to established plant proteins, such as soybeans. At present, *Pinus pumila* nut meal is only used as animal feed and has not been developed in the food industry, mainly due to the low protein extraction rate and associated problems with stability, texture, nutritional, and sensory aspects. The utilization of *Pinus pumila* nut proteins remains in its infancy due to the absence of structural properties, such as secondary structure ratios, hydrophobic interactions, disulfide bonds, ionic bonds, and other amino acid side‐chain interactions, and the structure of subunits.

The functional properties of plant proteins are highly contingent upon the extraction method employed (Ma et al. 2022). The conventional extraction method, performed at 40°C–50°C and under alkaline conditions, results in a high protein yield, but also low quality (due to the racemisation of amino acids and the loss of essential amino acids) and poor functional properties (Amagliani et al. 2017), and co‐extraction with phenolic compounds, which tended to cause color browning (Su et al. 2021). Discharging large quantities of lye can also be harmful to flora and fauna and is not conducive to environmental safety (Hewage et al. 2022). Therefore, there is a preference for associating novel green technologies to obtain highly functional plant proteins. Low‐power ultrasound (< 400 W) is a novel green technology (Pan‐utai et al. 2023; Zhao, Wang, et al. 2023). The cavitation shear force generated during the ultrasound process has the potential to break non‐covalent bonds, thereby increasing protein yield and functional properties (Qiu et al. 2023). Low‐power ultrasound also avoids the problems of localized high temperatures and excessive damage to plant cells associated with high‐power ultrasound (Zhao, Liu, and Xue (2023)). The extraction of plant proteins by proteases is mainly based on their degradation and modification of macromolecular proteins to make them soluble with higher recovery and purity (Zheng et al. 2024). Ultrasound‐assisted enzymatic extraction has emerged as a novel extraction method that can improve the efficiency of solvent mass transfer while at the same time synergistically modifying the protein structure to achieve higher solubility and functional properties (Turker and Isleroglu 2024). At present, ultrasound‐assisted enzyme extraction is mainly used to improve the antioxidant activity of fats and oils, the yield of polysaccharides (Yang et al. 2017) and the palatability of fiber (Gao et al. 2024). To the best of our knowledge, there have been no studies conducted on the functional activity of *Pinus pumila* protein.

Studies have shown that ultrasound‐assisted enzyme extraction has significant advantages in the extraction of plant proteins. Ultrasound‐assisted alkaline protease significantly improved the yield and dispersion of hickory protein (Wang et al. 2021); ultrasound‐assisted *Bacillus subtilis* protease significantly improved the functional properties and biological activity of *Brosimum alicastrum Swartz* proteins (Suárez‐Hernández et al. 2024). Therefore, in order to promote the widespread use of *Pinus pumila* nut protein, a study was carried out to optimize the extraction process, characterize the functional properties, analyze the physicochemical properties, and evaluate the nutritional composition of *Pinus pumila* nut protein after ultrasound‐assisted enzymatic modification. This study provides a theoretical basis for the characterization, structural, and nutritional aspects of *Pinus pumila* nut protein and proposes a 200 W ultrasound‐assisted papain modification of *Pinus pumila* nut protein to increase the yield and improve the functional properties. This study provides a scientific basis for the use of *Pinus pumila* nut protein in the food industry: (1) plant‐based foods (veggie meat and dairy alternatives); (2) bakery products (nutritional fortification and water‐holding agents for air‐filled pastries); (3) special diets (high protein energy bars with amino acid balance); and (4) food additives (emulsifiers and stabilizers).

## 2 Materials and Methods

### 2.1 Materials

The following materials were used: *Pinus pumila* kernel (Ning'an North Region Jenqi Mountain Forest Foods Co.); Soybean oil (Jiusan Oils & Grains Industries Group Co. Ltd.); Papain‐200 U/mg (Meryer (Shanghai) Chemical Technology Co. Ltd.); Bradford Protein Concentration Assay Kits (Fia Biotechnology Stock Co. Ltd.); Ellman Reagent (Thermo Fisher Scientific Inc.); Sodium Dodecyl Sulfate (SDS), Glycine, β‐Mercaptoethanol, Bovine Serum Albumin‐whole fraction (Beijing Solarbio Science & Technology Co. Ltd.); Petroleum ether, Potassium Bromide (Sinopharm Chemical Reagent Co. Ltd).

### 2.2 Traditional *Pinus pumila* Nut Protein Extraction

Petroleum ether was used to degrease the crushed *Pinus pumila* kernel (HXD‐200 Chinese medicine crusher, Tianjin Hengxinda Science and Technology Co. Ltd.). When the petroleum ether was colorless for filtration, the precipitate was defatted *Pinus pumila* nut oil meal. The defatted *Pinus pumila* nut oil meal was combined with water in a 1:30 ratio (g/mL), extracted at 45°C and pH 7.0 for 2.0 h, and then centrifuged at 6790 × g for 20 min (Low Temperature Ultra‐High Speed Centrifuge H2050R Hunan Xiangyi Experimental Instrument Development Co.). The pH value of the supernatant was adjusted to 4.0 (pH meter PHS‐3E Shanghai Yidian Scientific Instrument Co.). The mixture was centrifuged again and the precipitate was adjusted to neutrality, dialyzed for 48.0 h (Dialysis bags MD 34 Biosharp), and lyophilized (LGJ‐50F Freeze dryer, Beijing Songyuan Huaxing Technology Development Co.) to obtain the traditional *Pinus pumila* nut protein, named as TP.

### 2.3 Single‐Factor Experiment on Ultrasound‐Assisted Papain Extraction of *Pinus pumila* Nut Protein

The defatted *Pinus pumila* nut oil meal was mixed with distilled water and ultrasonicated at room temperature (25°C) for 30 min at 40KHz, 200 W (SB‐5200D, Ultrasonic Cleaner, NIingbo Scientz Biotechnology Co., LTD), followed by adjusting the pH value to 7.0. The enzymatic single‐factor experiments were conducted under the following conditions: solid–liquid ratio of 0.12 to 0.01; temperatures of 25°C to 75°C; enzyme dosages of 1000 to 12,500 U/g; hydrolysis time of 1.0 h to 3.5 h. After inactivating the enzyme in a boiling water bath for a period of 15 min, the solution was concentrated using a vacuum rotary evaporator (R1005 Zhengzhou Great Wall Science, Industry and Trade Co.). The remaining steps were carried out according to the established protocol for TP preparation. The ultrasound‐assisted papain‐modified *Pinus pumila* nut protein was designated as PUEP.

### 2.4 Response Surface Methodology to Optimize Extraction

Based on the outcomes of the single‐factor experiments, three significant factors were identified: the solid–liquid ratio (A), hydrolysis time (B), and enzyme dosage (C). RSM (Design‐Expert 12, Stat‐Ease Inc.) was used to further analyze and optimize the interaction between factors. The enzymatic temperature was selected to be 55°C for the subsequent experiments. The determination of high and low level values was achieved through the implementation of steepest climb experiments. The actual and coded values of the independent variables in the RSM are shown in Table 1.

**TABLE 1:** Actual encoded values of each factor in RSM.

	−1	+1	0
Hydrolysis time (h)	2.12	2.20	2.16
Solid–liquid ratio	0.016	0.010	0.013
Enzyme dosage (U/g)	3751	4251	4001

### 2.5 Solubility

The protein solution with a concentration of 1 mg/mL was configured using water, salt, acid, and alkali. The protein solution after ultrasonic dispersion was determined using a spectrophotometer (T6, Beijing Pulse Analyzer General Instrument Co.) using the Coomassie blue staining (Sedmak and Grossberg 1977). The protein solubility was calculated in accordance with the following formula:

(1) $$\text{Solubility}\;\left(\%\right)=C_{1}/C_{0}\times 100\%$$

In the formula: *C*_1_ represents the actual proteolytic concentration; *C*_0_ denotes the configured protein concentration (1 mg/mL).

### 2.6 Emulsification Activity Index (EAI) and Emulsion Stability Index (ESI)

A protein concentration of 5 mg/mL was prepared and combined with soybean oil and homogenized using a high‐speed homogenizer (FJ200‐S, Shanghai Amperexperiment Technology Co.). At 0 and 10 min after homogenization, the emulsion was added to a 0.1% (g/mL) SDS solution. The absorbance was then measured at 500 nm (Sun et al. 2024). EAI and ESI were calculated according to the following formula:

(2) $$\mathrm{EAI}\;\left(m^{2}/g\right)\;=\;2\times 2.303\times A_{0}\times \mathrm{DF}/\left[10000C\;\times \;\phi \;\times \;\left(1-\theta \right)\right]$$

(3) $$\mathrm{ESI}\;\left(\min\right)\;=\;10A_{0}/\left(A_{0}\;-\;A_{10}\right)$$

In this formula, DF is the dilution factor (100); *C* is the initial protein concentration (mg/mL); φ is the optical range (0.01 m); *θ* is the volume fraction of oil used to form the emulsion (0.9).

### 2.7 Foaming Capacity and Foam Stability

A sample solution of 2 mg/mL was prepared by dispersing the protein in water, salt, acid, and base. The FC and FS were calculated using the following formula (Xie et al. 2023):

(4) $$\mathrm{FC}\;\left(\%\right)\;=\;\left(V_{1}\;-\;V_{0}\right)/V_{0}\times 100\%$$

(5) $$\mathrm{FS}\;\left(\%\right)\;=\;F_{10}/F_{0}\times 100\%$$

Here, *V*_1_ is the total volume after homogenization (mL); *V*_0_ is the initial volume of the solution (mL); *F*_0_ is the foam volume after homogenization (mL); *F*_10_ is the foam volume after 10 min (mL).

### 2.8 Water Holding Capability and Oil Holding Capability

The WHC/OHC of the protein was calculated using the following formula (Wang et al. 2023):

(6) $$\mathrm{WHC}/\mathrm{OHC}\;\left(g/g\right)\;=\;\left(W_{2}\;-\;W_{1}\;-\;W_{0}\right)/W_{1}$$

In this formula, *W*_*0*_ is the mass of the centrifuge tube (*g*); *W*_*1*_ is the mass of the protein (g); *W*_*2*_ is the total mass of the centrifuge tube and precipitate (g)

### 2.9 Surface Hydrophobicity

ANS (8.0 mmol/L) and protein solution (1 mg/mL) were ultrasonically dispersed (Yan et al. 2021). The fluorescence intensity of the solution was measured at an excitation wavelength of 374 nm and an emission wavelength of 485 nm by microplate reader (550 Bio‐Rad, USA). A linear fit was performed with the sample concentration as the horizontal coordinate and the sample fluorescence intensity as the vertical coordinate, and the slope is the surface hydrophobicity (H_0_).

### 2.10 Particle Size and Zeta Potential

Protein solutions (1 mg/mL) were prepared (Bing et al. 2024), then centrifuged, and the supernatant was filtered through a 0.22 μm membrane and then analyzed for particle size, zeta potential, and polymer dispersity index (PDI) using the Zetasizer Nano ZS (Malvern Panalytical, UK).

### 2.11 TG‐DSC Simultaneous Thermal Analysis

The tests were carried out in N_2_ with a temperature range of 30°C to 300°C and a temperature rise rate of 5°C/min, and were determined using a simultaneous thermal analyzer (Netzsch STA 449 F3, Germany).

### 2.12 SDS‐Page

The protein concentration was set at 2 mg/mL, and the molecular weights of the proteins were calculated based on the mobility of the standard proteins (Guo, Wang, et al. 2023).

### 2.13 Color Difference Analysis

The brightness (L*) and red/green (a*) or blue/yellow (b*) color bias of TP and PUEP and the color difference (ΔE) between them were measured using a colorimeter (SC‐20, Hangzhou Chroma Technology Co.). ΔE was calculated according to the following formula:

(7) $$\mathrm{\Delta E}\;=\;\sqrt{{\left(L_{1}\;-\;L_{2}\right)}^{2}\;+\;{\left(a{*}_{1}\;-\;a{*}_{2}\right)}^{2}\;+\;{\left(b{*}_{1}\;-\;b{*}_{2}\right)}^{2}}$$

In this formula, *L*_1_ and *L*_2_ are the brightness of the TP and PUEP; a*_1_ and a*_2_ are the red/green color biases of the TP and PUEP; b*_1_ and b*_2_ are the blue/yellow color biases of the TP and PUEP.

### 2.14 Sulfhydryl Group and Disulfide Bond Content

PUEP was dissolved in 10 mL of 8 mol/L urea Tris‐Gly buffer. Free sulfhydryl: protein solution, Tris‐Gly buffer, and Ellman's reagent (DTNB) were added in a ratio of 1:4:0.05, and the Abs was measured at 412 nm after 5 min. Total sulfhydryl content: protein solution, β‐mercaptoethanol and Tris‐Gly buffer were added in a ratio of 1:0.05:4. 20 mL of 12% TCA was added for 1 h. After centrifugation of the solution, the precipitate was dissolved in 10 mL of Tris‐Gly buffer and then added to Elman's reagent at a 100:1 ratio and reacted for 5 min. Solution UV absorbance was measured at 412 nm. Sulfhydryl group and disulfide bond content were calculated according to the following formula:

(8) $$\text{Free sulfhydryl}/\left(\text{μmol}/g\right)=73.53\times A_{412}\times D/C$$

(9) $$\text{Disulfide bond}/\left(\text{μmol}/g\right)=\left(-\mathrm{SH}-\text{Free Sulfhydryl}\right)/2$$

In this formula, *C* is the protein concentration (mg/mL); *D* is the dilution constant (free sulfhydryl‐5; total sulfhydryl−10); 73.53 is the molar absorption coefficient.

### 2.15 Determination of Fourier Transform Infrared Spectrum

Protein samples (2 mg) were taken and 1:200 (w/w) of KBr was added in an onyx mortar and thoroughly ground, mixed homogeneously, and pressed into tablets (Gorlov et al. 2018), and then scanned with an infrared spectrometer (Nicolet iS 10, Thermo Fisher Scientific, USA) in the frequency range of 400 to 4000 cm^−1^ for 128 scans.

### 2.16 Determination of Ultraviolet–Visible Absorption Spectrum

Protein samples (1 mg/mL) were prepared in double‐distilled water, passed through a 0.22 μm filter membrane, and then scanned at full wavelength at 230–600 nm using a multimode plate reader (Infinite M200 pro, Tecan (Shanghai) Trading Co. Ltd., Switzerland). The scanning interval was 1 nm/s.

### 2.17 Morphological Analysis (SEM)

The proteins were sprayed with gold treatment and subjected to secondary electron examination on conductive adhesive at magnifications of 200×, 500×, 1000×, 2000×, and HV = 5.00 KV using SEM (FEI‐NOVA, NANOSEM 230, USA).

### 2.18 Analysis of Amino Acid Content

The protein sample (100 mg) was hydrolysed with 8 mL of 6 M HCl under vacuum for 24 h. The sample was neutralized by the addition of 4.8 mL of 10 M NaOH, diluted to 25 mL, and filtered (Sá et al. 2024). The protein amino acid content was determined using an amino acid analyzer (MembraPure A300, German).

### 2.19 Statistical Analysis

Results are expressed as mean ± standard deviation. One‐way analyses of variance (ANOVA) were performed using SPSS (version 20.0, USA) to determine the significance of differences between variable means at *p < 0.05*. Significant differences between groups are labeled in lower case (maximum mean group labeled a). Origin 2022 was used to create experimental graphs.

## 3 Results and Discussion

### 3.1 Single Factor Experiment

**FIGURE 1:**
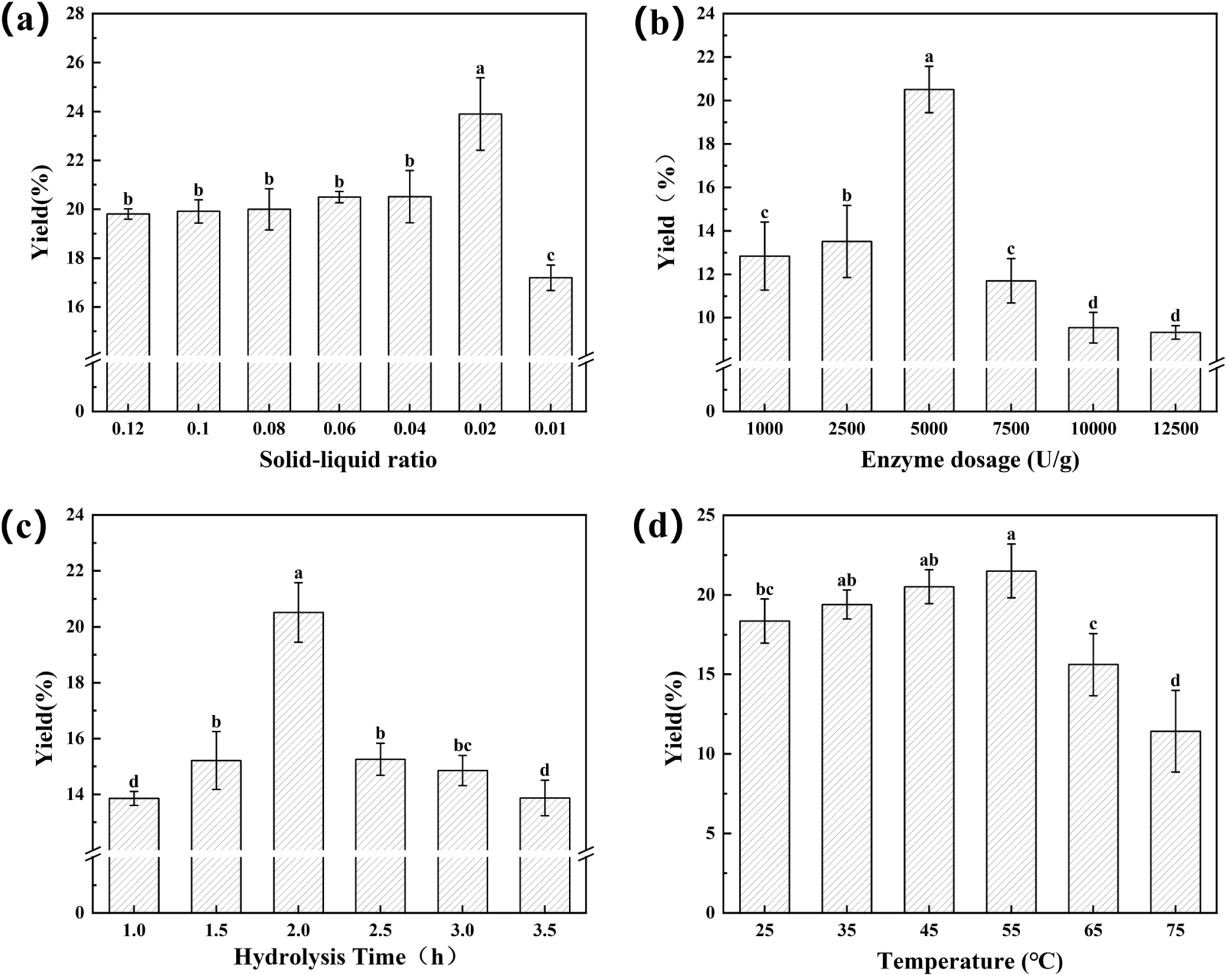
Effect of ultrasound‐assisted enzymatic extraction conditions on the yield of PUEP: (a) Solid–liquid ratio; (b) Enzyme dosage; (c) Hydrolysis time; (d) Temperature.

Proteases can easily increase yield by hydrolysing some large proteins, reducing the molar mass and accelerating the release of proteins in the plant cell wall, while preventing the formation of internal protein complexes to increase the content of soluble proteins (Hadidi et al. 2023). As illustrated in Figure 1a, the yield of PUEP exhibited a gradual and insignificant increase with the reduction in the solid–liquid ratio. The protein yield reached its maximum at a solid–liquid ratio of 0.02, with a notable difference in outcome. This was attributed to a gradual reduction in the viscosity of the extraction solution and an increase in the molecular diffusion rate and the enhanced contact area of the enzyme with the substrate. The continuation of an increase in the solid–liquid ratio resulted in a notable reduction in protein yield, attributed to the low concentration of the solution, which consequently led to a reduction in enzyme‐substrate contact. As illustrated in Figure 1b, the positive correlation between the enzyme dosage in the range of 1000–5000 U/g and the yield of PUEP can be attributed to the insufficient interaction between the enzyme and the substrate when the enzyme dosage was low. An increase in the enzyme dosage could lead to the complete enzyme digestion reaction. The highest yield of PUEP was obtained at an enzyme dosage of 5000 U/g. As the enzyme dosage increased, the yield steadily decreased, which was attributed to the fact that the enzyme dosage was too high, resulting in saturation of the substrate and the enzyme binding site. It was hypothesized that the oversaturated protease might interact with or even hydrolyse the extracted target proteins, thereby reducing the yield. As illustrated in Figure 1c, the protein yield demonstrated a notable increase with the prolongation of the enzymatic hydrolysis time, reaching a peak (20.51%) at 2.0 h of hydrolysis. This observed phenomenon can be attributed to the prolonged enzymatic hydrolysis time, which facilitated the further hydrolysis of the disrupted pine kernel cell wall by papain, resulting in the dissolution of protein and a subsequent enhancement in yield. Protein yield decreased significantly with prolonged hydrolysis time because prolonged hydrolysis leads to coagulation of soluble proteins with other components (e.g., phytates) reducing the yield (Eromosele et al. 2008), while the extracted target proteins may be over‐hydrolysed by the enzyme. As illustrated in Figure 1d, the protein yield exhibited an increasing trend with increasing enzymatic temperature, reaching a maximum value (21.32%) at 55°C. This is attributed to the temperature reaching the optimal temperature for enzymatic reaction. Conversely, the protein yield exhibited a gradual decline with increasing enzymatic temperature. This is attributed to the denaturation and inactivation of proteins under high temperature conditions.

### 3.2 PUEP Extraction Response Surfaces Analysis (Box–Behnken)

A Box–Behnken experiment typically comprises 12 analytical factorization tests and 5 central tests. The design and results of this experiment are presented in Table 2 below. Here, A represents the solid–liquid ratio, B denotes hydrolysis time, and C is the enzyme dosage.

**TABLE 2:** Box–Behnken design and response results.

Std	RUN	A	B	C	Yield (%)
12	1	0	0	0	29.48
5	2	0	0	0	29.29
6	3	−1	0	1	26.13
10	4	−1	1	0	26.68
7	5	−1	0	−1	27.32
8	6	1	0	1	25.88
11	7	−1	−1	0	25.76
14	8	1	0	−1	24.83
15	9	1	−1	0	26.58
1	10	0	0	0	29.08
17	11	0	0	0	28.82
16	12	0	−1	1	25.56
9	13	0	v1	−1	27.59
3	14	0	1	1	26.89
2	15	0	1	−1	25.14
13	16	0	0	0	29.36
4	17	1	1	0	24.86

The data were subjected to analysis using RSREG (Response Surface Regression) of Design‐Expert 12, and the results of the regression analysis and ANOVA are presented in Table 3 below. The ANOVA model with *F* = 50.39, *p* < 0.0001 indicates that the experimental model is highly significant and the experimental design plan is reliable and statistically significant. The lack of fit *p =* 0.2651 > 0.05, indicating that the lack of fit of the regression model is not significant. The correlation coefficient of the model *R* ^2^ = 0.9848, *R*_adj_ = 0.9653 indicates that the model is well fitted and that the predicted values have a strong correlation with the actual values, which explains 98.48% of the variation in the response values. This model can therefore be used to predict the optimal process for PUEP extraction. The formula used to predict the PUEP yield is as follows:

**TABLE 3:** Box–Behnken design on the variance analysis and *p*‐value.

Source	Sum of squares	df	Mean square	*F*‐value	*p*‐value	Significance
Model	43.05	9	4.78	50.39	< 0.0001	※※※
A	1.75	1	1.75	18.42	0.0036	※※
B	0.4608	1	0.4608	4.85	0.0634	
C	0.022	1	0.022	0.2323	0.6445	
AB	1.74	1	1.74	18.36	0.0036	※※
AC	1.25	1	1.25	13.22	0.0083	※
BC	3.57	1	3.57	37.63	0.0005	※※
A^2^	12.83	1	12.83	135.15	< 0.0001	※※※
B^2^	9.35	1	9.35	98.55	< 0.0001	※※※
C^2^	8.5	1	8.5	89.51	< 0.0001	※※※
Residual	0.6644	7	0.0949			
Lack of fit	0.3937	3	0.1312	1.94	0.2651	
Pure error	0.2707	4	0.0677			
Cor total	43.71	16				

**FIGURE 2:**
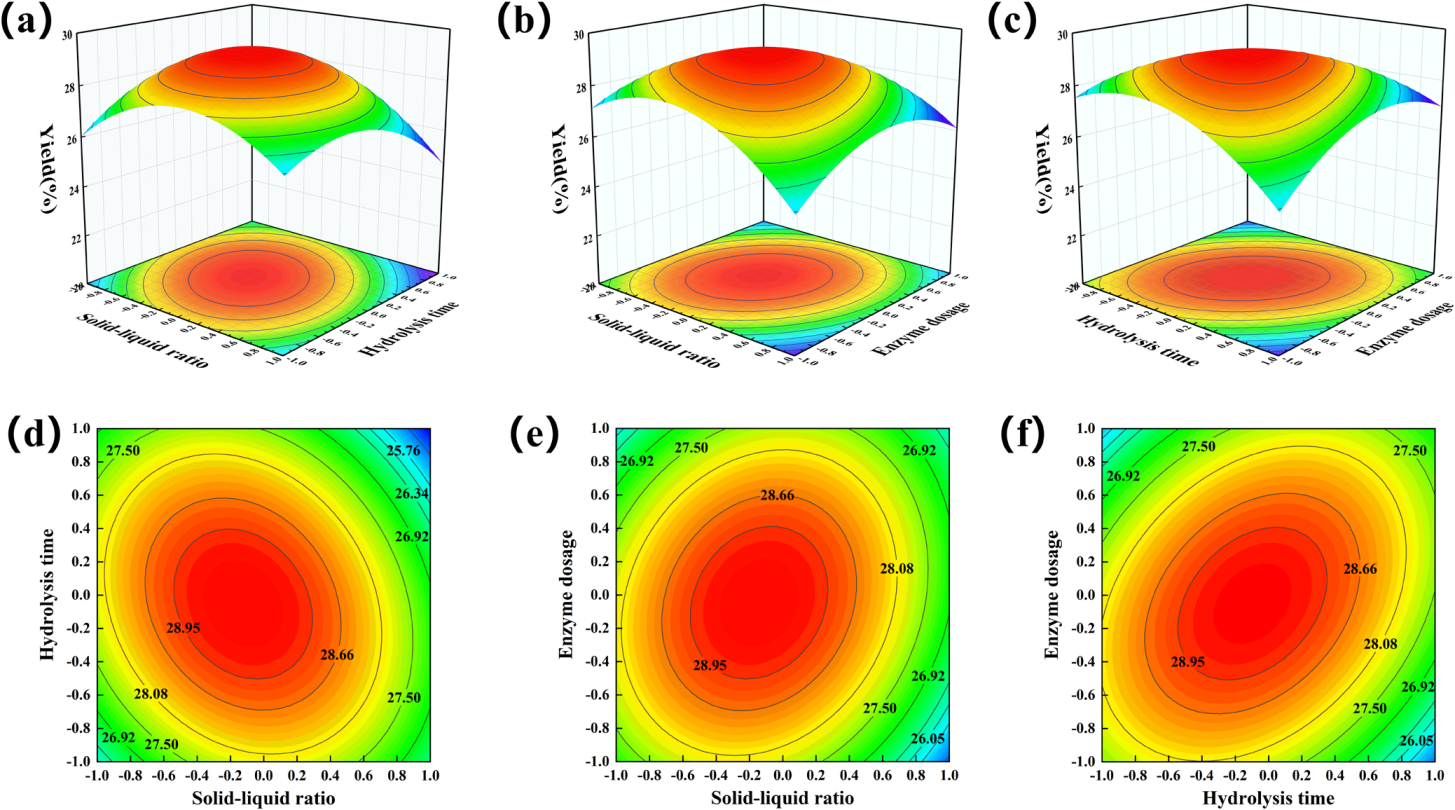
Interaction diagram: (a) Solid–liquid ratio and Hydrolysis Time; (b) Solid–liquid ratio and Enzyme dosage; (c) Hydrolysis Time and Enzyme dosage. Contour graph: (d) Solid–liquid ratio and Hydrolysis Time; (e) Solid–liquid ratio and Enzyme dosage; (f) Hydrolysis Time and Enzyme dosage.

*Y* = 29.206 − 0.4675A − 0.24B − 0.0525C − 0.66AB + 0.56 AC + 0.945 bc − 1.7455A^2^‐1.4905B^2^ − 1.4205C^2^. *p*‐value analysis of the coefficients of the model shows that the *p*‐values of factors A, AB, and AC are < 0.01, indicating that the effects of hydrolysis time, enzyme dosage, and solid–liquid ratio on the yield of PUEP are highly significant. The RSM plots of the factors affecting the yield of PUEP are shown in Figure 2, which shows that there are obvious interactions between the three factors selected in the experiment, rather than a purely linear relationship. From Figure 2 and the *F*‐values in Table 3, it can be seen that the most important factors affecting the PUEP yield are: solid–liquid ratio > hydrolysis time > enzyme dosage.

The model predicted the optimal extraction process parameters for PUEP as follows: solid–liquid ratio of 1:74.66 (g/mL), hydrolysis time of 2.157 h, enzyme dosage of 3984 U/g, and theoretically predicted yield: 29.247% at an enzyme temperature of 55°C. Based on the consideration of the actual experimental conditions, the best extraction conditions were determined to be a solid–liquid ratio of 1:75 (g/mL), a hydrolysis time of 2.15 h, and an enzyme dosage of 3980 U/g. The actual yield of PUEP under these conditions was verified by three parallel experiments to be 28.89%, and the extraction rate was up to 83.72%, which was not significantly different from the theoretical value, indicating that the model can be used to predict the PUEP extraction. It is notable that the TP yield was 8.587%, and the PUEP yield increased by approximately a factor of 3.5. The yield of ultrasound‐assisted enzymatic extraction of *Pinus pumila* nut protein was found to be higher than that of ultrasound‐assisted vacuum alkaline extraction. Furthermore, the extraction environment is environmentally friendly, which suggests that PUEP has the potential for application in the food industry.

### 3.3 Functional Properties of Proteins

#### 3.3.1 Solubility

The water solubility of proteins is a crucial factor in determining their functional properties. Table 4 shows the solubility of PUEP and TP. In a salt solution, the solubility of TP and PUEP was found to be significantly increased (20.05% and 27.143%), which was attributed to the increase in the surface charge of the protein in the solution rich in salt ions, and the enhanced electrostatic repulsive effect. The improved solubility of the protein in 0.01 M lye is due to the fact that in an alkaline environment, electrostatic repulsion between protein molecules is enhanced, facilitating the unfolding of its structure, enhancing hydration, and improving its dispersion (Xu et al. 2023). The PUEP exhibited the highest solubility (52.94%) in lye, which was significantly higher than that of TP, which was close to Korean pine protein after roasting treatment (Liu et al. 2022). This phenomenon can be attributed to the ultrasound‐assisted enzymatic extraction resulting in the conversion of β‐sheet structures into β‐turns in the secondary structure of the protein, as proteolysis increases with decreasing relative protein β‐sheet content (Mune Mune et al. 2018). As localized exposure of hydrophobic groups leads to conformational relaxation (Yue et al. 2024), the overall solubility of PUEP was higher than that of TP, suggesting that the increase in solubility is a result of the modified modification of natural proteins.

**TABLE 4:** Functional characteristics of TP and PUEP.

		Solubility (%)	EAI (m^2^/g)	ESI (min)	FC (%)	FS (%)
TP	H_2_O	1.10 ± 0.07^g^	4.86 ± 0.01^d^	67.74 ± 10.67^b^	3.00 ± 0.71^h^	50.04 ± 4.19^e^
	0.1 M PBS	20.05 ± 1.18^f^	5.25 ± 0.17^c^	71.85 ± 7.25^b^	43.35 ± 0.69^b^	58.99 ± 1.90^d^
	0.01 M HCl	32.65 ± 0.92^d^	2.45 ± 0.01^g^	53.94 ± 0.89^c^	19.97 ± 1.37^e^	73.00 ± 3.29^b^
	0.01 M NaOH	44.11 ± 1.92^b^	4.63 ± 0.04^e^	51.07 ± 2.59^c^	13.73 ± 1.73^g^	53.59 ± 4.19^e^
PUEP	H_2_O	0.89 ± 0.14^g^	5.26 ± 0.15^c^	66.77 ± 8.24^b^	15.32 ± 0.86^f^	73.87 ± 4.15^b^
	0.1 M PBS	27.14 ± 1.52^e^	6.28 ± 0.26^b^	102.74 ± 6.18^a^	49.45 ± 1.24^a^	67.30 ± 0.53^c^
	0.01 M HCl	35.80 ± 1.24^c^	3.76 ± 0.02^f^	68.71 ± 4.32^b^	25.34 ± 0.47^c^	79.54 ± 0.54^a^
	0.01 M NaOH	52.94 ± 1.98^a^	6.53 ± 0.01^a^	64.69 ± 2.69^b^	21.47 ± 2.08^d^	60.92 ± 4.36^d^

*Note:* Data in this table are analyzed by one‐way ANOVA test, and different letters in the same column indicate significant differences (*p* < 0.05).

#### 3.3.2 Emulsifying Activity Index (EAI) and Emulsifying Stability Index (ESI)

The fundamental functions of protein in food and beverage production are emulsibility and solubility (Yue et al. 2024). The general trend of emulsification of TP and PUEP is analogous to that of solubility, and the ESI of PUEP was significantly higher than that of TP, which has been attributed to the dispersion of large protein clusters by ultrasound, the increase in the proportion of small soluble proteins (Tian et al. 2024), the increased exposure of hydrophobic groups of PUEP (Dai et al. 2024), and the denaturation of some of the proteins, the change in molecular structure, and the exposure of hydrophilic groups by the enzyme shear, which together resulted in high adsorption of PUEP at the water–oil interface. As illustrated in Table 4, the emulsibility of *Pinus pumila* nut protein in lye was found to be the most elevated, with PUEP (6.525 m^2^/g) increasing by 40.92% in comparison to TP. This was attributed to the unfolding of its structure and the exposure of hydrophobic residues, which were conducive to the formation of a water–oil interface. PUEP demonstrated excellent emulsifying properties (6.280 m^2^/g) and emulsifying stability (102.74 min) in saline solution, twice that of alkaline extracted *Pinus pinea* nut protein (Benzitoune et al. 2022).

#### 3.3.3 Foaming Capacity (FC) and Foam Stability (FS)

Due to their hydrophobicity, proteins can rapidly adsorb and form an elastic adsorbent layer at the air‐water interface, conferring them with excellent FC and FS (Farid et al. 2023). As illustrated in Table 4, the PUEP exhibits enhanced foaming capacity (FC) and foaming stability (FS) in comparison to TP. The FC of *Pinus pumila* nut protein in different solvents was as follows: 0.1 M PBS > 0.01 M HCl > 0.01 M NaOH > H_2_O. The FC of PUEP was significantly increased by 14.07% (FS = 49.45%) in salt solution, 410.6% in water, 26.89% in acid solution, and 56.37% in alkaline solution compared to TP. The addition of low concentrations of salt ions was found to favor protein foaming and to produce similar results in plasma‐extracted oat proteins (Eazhumalai et al. 2023) and water‐extracted red pine protein. The FS of *Pinus pumila* nut protein in different solvents was as follows: 0.01 M HCl > 0.1 M PBS > 0.01 M NaOH > H_2_O. The PUEP exhibited an excellent FS (79.54%), which was significantly higher than that of TP in 0.01 M HCl, which can be attributed to the improved solubility and reduced surface tension following the ultrasonic treatment, resulting in the formation of more clearly delineated hydrophobic and hydrophilic regions, facilitating the rapid movement of the PUEP towards the interface, thereby producing a more stable foam. Compared to other plant proteins (Karimi et al. 2024; Wu et al. 2023), *Pinus pumila* nut protein has high FC and FS. However, it is advisable to avoid using this protein in water as much as possible, and its use in foods should be dissolved in salt or low pH liquids.

#### 3.3.4 Water and Oil Holding Capacity (WHC/OHC)

**FIGURE 3:**
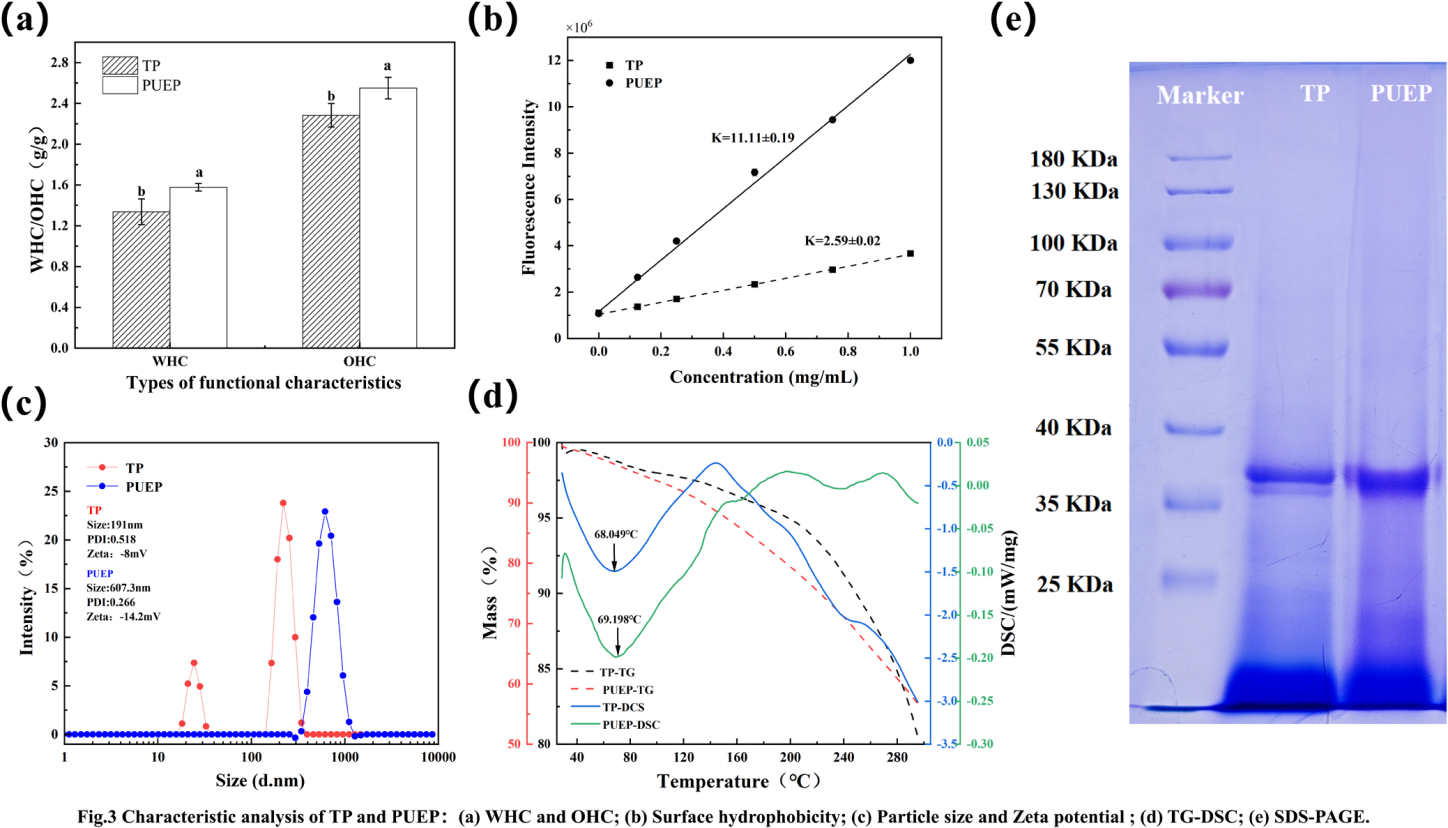
Characteristic analysis of TP and PUEP: (a) WHC and OHC; (b) Surface hydrophobicity; (c) Particle size and Zeta potential; (d) TG‐DSC; (e) SDS‐PAGE.

WHC and OHC play a key role in the preparation of viscous foods and are essential for the formation of stable gels (Gharibzahedi and Smith 2020). TP has a WHC of 1.34 g/g and an OHC of 2.15 g/g, and PUEP has a WHC of 1.56 g/g and an OHC of 2.47 g/g, as shown in Figure 3a. Ultrasound‐assisted enzymatic modification significantly increased the WHC and OHC of *Pinus pumila* nut protein, and the OHC of PUEP was 1.42 times higher than that of pea protein prepared under low pressure conditions (D'Alessio et al. 2023), and 1.36 times higher than that of rice hull protein (Guo, Liu, et al. 2023). This is due to the fact that ultrasound exposes more polar groups and hydrophobic side chains of the protein (Laing et al. 2023), while enzymatic extraction causes a relaxation of the spatial conformation of PUEP, providing a larger specific surface area and more lipophilic sites, which in turn bind more oil, leading to an increase in OHC. Compared to soy protein (Zhang et al. 2023), *Pinus pumila* nut protein is rich in hydrophobic amino acids (30.12 g/100 g), which gives it excellent WHC/OHC, favoring its use in plant‐based meats. Complete denaturation of the protein leads to a reduction in WHC/OHC, demonstrating that the modified conditions of this experiment are appropriate.

### 3.4 Physical and Chemical Properties of the Protein

#### 3.4.1 Surface Hydrophobicity (H_0_ )

H_0_ is an important parameter for assessing changes in the surface of proteins in contact with polar aqueous environments, reflecting the number of hydrophobic groups in proteins, spatial conformation, and possible interfacial functions (Wu et al. 2023). As shown in Figure 3b, the H_0_ of PUEP was significantly increased compared to that of TP, which is consistent with the results of ultrasound/ultrasound‐assisted enzymatic digestion of extracted sunflower protein, wheat germ protein, zein, and alginate protein (Dabbour, Alenyorege, et al. 2020; Lian et al. 2021; Ren et al. 2018; Yang et al. 2017). This may be due to the unfolded conformation of PUEP and the dispersion of the aggregates, exposing the hydrophobic groups to the surface (Zhao, Wang, et al. 2023), which is one of the reasons for the larger particle size. As the stability of natural proteins is maintained by hydrophobic interactions, the H_0_ of proteins usually increases after modification (Helmick et al. 2023). H_0_ prevents protein aggregation and helps to create a stable emulsion system, that is, surface hydrophobicity is positively correlated with protein emulsibility (Tang et al. 2023).

#### 3.4.2 Particle Size and Zeta Potential

The particle size of PUEP (607.3 nm) increased 2.17‐fold compared to TP (191 nm) and was close to that of red pine kernel protein, as shown in Figure 3c. The PDI value of the PUEP solution is 0.266 < 0.518, which is more stable compared to TP due to the high surface hydrophobicity that causes the charged protein particles to interact with water as dipole molecules. Similar to the findings of CHEN et al., ultrasound at 300 W or lower treatment power can loosen the protein structure, exposing the active groups that interact with each other, resulting in protein aggregation and increased particle size (Chen and Zhang 2023). The zeta potential of *Pinus pumila* nut proteins suggests that they are negatively charged at the interface, mainly due to the dissociation and protonation of amino acids on the protein surface in water. The absolute increase in the zeta potential of PUEP (−14.2 mV) compared to TP (−8.0 mV) indicates that the reduction in the degree of aggregation is beneficial to the increase in its functional properties, following the same trend as for enzymatically prepared pea, rice, hemp, and oat proteins (Shahbal et al. 2023).

#### 3.4.3 Protein Thermogravimetric Analysis (TG‐DSC)

Simultaneous thermal analysis (TG‐DSC) captures mass changes and thermal effects of proteins simultaneously to identify the physical and chemical processes taking place (Hu et al. 2023). The results of the thermogravimetric analyses of TP and PUEP are shown in Figure 3d, and the same trend of mass loss was observed for both in the temperature range tested (25°C–300°C), where the first degradation of the protein took place; no rupture of the chemical bond occurred and no inflection point appeared, suggesting that the mass loss in this range was mainly caused by moisture. Mass loss (%) = 5.0 is commonly used to assess thermal stability. The lower structural thermal stability of PUEP may be due to the accelerated denaturation of the protein by the ultrasound‐assisted enzyme treatment, resulting in a more relaxed structure. The higher the ΔH in the DSC curve, the stronger the thermal stability of the protein, and the ΔH value of TP was higher than that of PUEP, which was further confirmed by its strong thermal stability. Both TP and PUEP produced distinct DSC thermal absorption peaks without significant mass loss at around 70°C; this time, the proteins underwent melt denaturation, close to the denaturation temperature of pea proteins (Kuang et al. 2023). The higher mass loss and enthalpy of denaturation of PUEP confirms the reduced thermal stability, which may be due to unnatural extraction methods that break chemical bonds and alter protein structure.

#### 3.4.4 SDS‐Page

Characteristics such as molecular weight, types of specific subunits, and protein ratios are important factors in determining the use of plant proteins. The molecular weight distribution of *Pinus pumila* nut protein was determined by SDS‐PAGE. The molecular weight of the major subunit of the protein is represented by the darkest and clearest band in the SDS‐PAGE plot (Li et al. 2024). As shown in Figure 3e, the major subunits of *Pinus pumila* nut protein appeared at 30 –40 KDa, indicating that the total molecular weight of the *Pinus pumila* nut protein was concentrated here and that the ultrasound‐assisted enzyme had a weak effect on the molecular weight. The molecular weights of TP were mainly 36.15, 34.10, 24.88, 21.62, and 19.70 KDa, compared to other tree nut proteins, *Pinus pumila* nut protein has a lower molecular weight and may have higher absorption and biological activity. The molecular weights of PUEP were 34.50, 26.37, 21.57, and 19.02 KDa. PUEP macromolecular protein bands appeared blurred and weaker and contained more small molecule proteins, which is potentially due to enzymatic extraction degrading large molecules or reducing the extraction of high molecular weight subunits. The decrease in molecular weight of PUEP indicates a reduction in protein aggregation and loosening of the structure, which is consistent with the functional and physicochemical characterization of PUEP. It is worth noting that ultrasound has no significant effect on molecular weight, as the protein backbone can withstand the shear force generated by ultrasound (Chakravorty and Das 2023).

#### 3.4.5 Analysis of Color Differences

Color is an essential feature of food and plays an important role in increasing product acceptance and popularity (Xu et al. 2022). Consistent with most of the plant proteins (Özdemir et al. 2022), *Pinus pumila* nut protein has high L* values (82.33 ± 0.81) with a predominant white color; b* values (15.97 ± 0.27) > 0 show a yellow trend, a* values (1.98 ± 0.21) > 0 show a light red trend. PUEP showed no significant difference in L* and a* compared to TP, and a significant difference in b* values (TP‐26.52 ± 0.95) with a color shift from pale yellow to whitish gray, which could be attributed to the effect of ultrasonic shear opening the side chain structure of PUEP and causing a Maillard reaction that reduces the brightness and color of the sample. When ΔE is greater than 4.0, it usually means that there is a significant difference between the two measurements, visible to the naked eye. The ΔE between PUEP and TP is 11.31, which is a significant difference in color (Wright et al. 2009) and in most cases cannot be mixed in the food industry.

#### 3.4.6 Disulphide Bond and Sulfhydryl Content

Sulfhydryl groups play an important role in protein folding and stability and can reflect changes in the tertiary structure of proteins (Zhao et al. 2022), and disulfide bonds can improve the structural stability and function of proteins. PUEP has 13.87% lower total sulfhydryl content, 16.22% lower free sulfhydryl content, and 13.27% lower disulphide bond content than TP, suggesting that the tertiary structure of PUEP has been altered under unnatural conditions. This is consistent with changes in the sulfhydryl content of pea proteins extracted by different methods, which may be due to conformational deformation that exposes the internal‐SH group of the protein (Gao et al. 2020). Low‐power ultrasound of PUEP results in the unfolding of the protein subunits and the breaking of disulfide bonds, exposing the internal sulfhydryl groups (Jiang et al. 2022), significantly reducing the disulfide bond content and forming a more pliable conformation that enhances adsorption at the air‐water interface. The decrease in disulfide bond content indicates that the structure of PUEP aggregates is stretched, which corresponds to the increase in solubility of PUEP, whose molecular structure is likely to be more flexible and looser (Xu et al. 2023).

#### 3.4.7 Analysis of Fourier Transform Infrared Spectra

**FIGURE 4:**
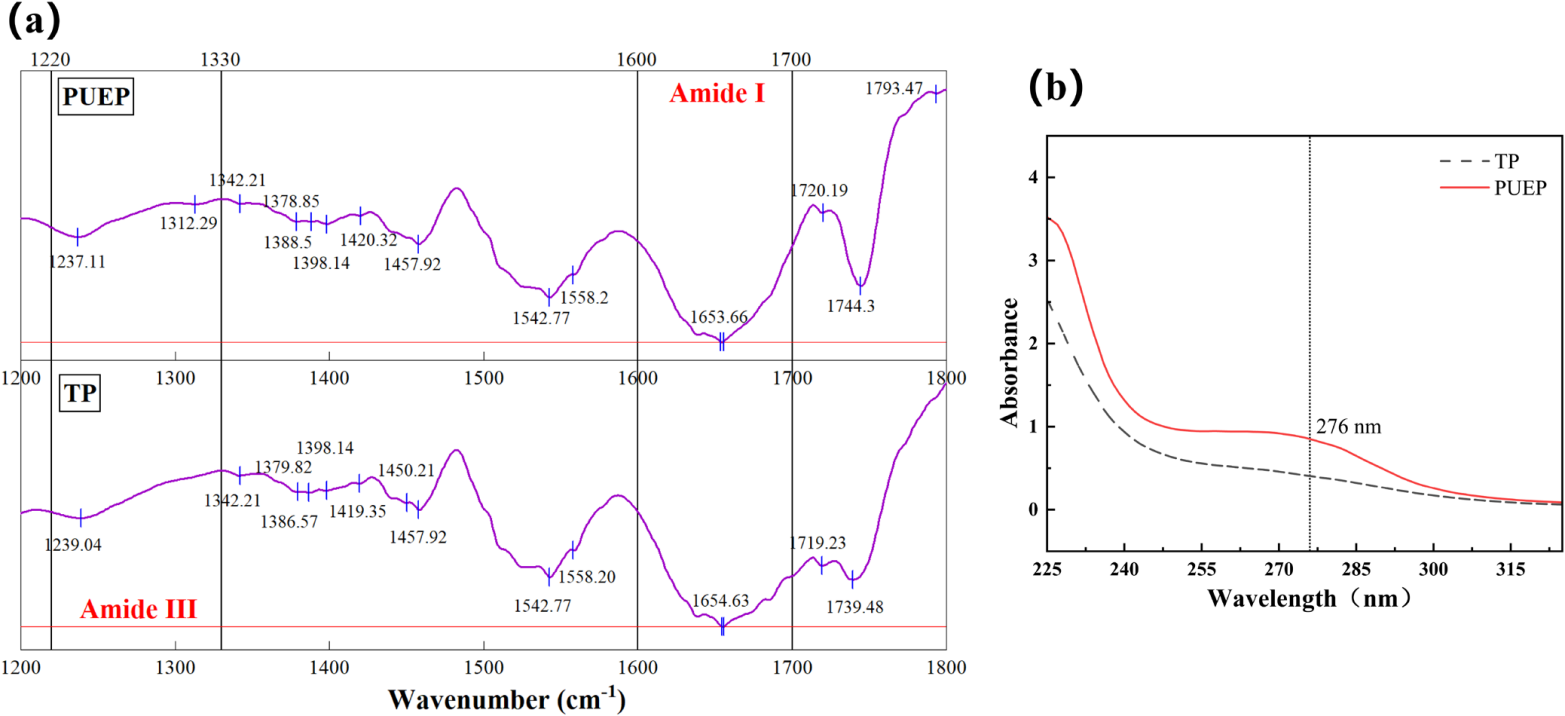
Characterization plots of PUEP and TP: (a) Fourier transform infrared (FTIR) spectrum; (b) UV–visible spectrum.

The amide I region (1700–1600 cm^−1^) of Fourier transform infrared spectroscopy is the most sensitive spectral region for the secondary structure components of proteins, with approximately 80% being caused by C = O stretching vibrations in the peptide bonds (Kong and Yu 2007). The amide III band (1330 ~ 1220 cm^−1^) can complement the distinction between the α‐helix and the random coil two structures in the secondary structure of proteins. As shown in Figure 4a, there are obvious β‐sheet absorption peaks near 1237 cm^−1^ for both TP and PUEP in the amide III band, and an α‐helical absorption peak near 1312 cm^−1^ for PUEP. In the amide I band, both TP and PUEP have β‐sheet absorption peaks at 1637 cm^−1^ and α‐helical absorption peaks at 1654 cm^−1^, while PUEP has a weakened absorption peak at 1678 and 1686 cm^−1^. The relative content of protein secondary structure was obtained after FSD curve fitting, baseline correction, Fourier deconvolution, and second order derivation. The decrease in the α‐helix content of PUEP compared to TP may be due to the unfolding of the α‐helix region of the protein by the combined effect of sonication and enzymatic degradation, thereby altering the functional properties of the protein (Umego et al. 2021). The β‐sheet content of PUEP was reduced by 45.58% compared to TP, which was attributed to the interference of sonication during the extraction process, resulting in the disruption of hydrogen bonds formed by alternating peptide bonds between adjacent peptide chains (Ren et al. 2022). The relative content of β‐turns of PUEP increases, which is negatively correlated with hydrophobicity (Shu and Yong 2017), consistent with the experimental results of high solubility and low disulfide bond content of PUEP.

#### 3.4.8 Analysis of UV–Visible Spectra

The electronic absorption spectra of proteins are mainly generated in the UV region (180–320 nm) due to the characteristic absorption of peptide bonds (~190 nm) and aromatic amino acids (~270 nm) (Mandal et al. 2017). As shown in Figure 4b, the overall light absorption intensity of PUEP was significantly improved compared to TP at the same concentration, which can be attributed to the fact that ultrasound buries the intermolecular interactions, causing the molecules to unfold and expose more protein clusters, leading to an increase in absorption intensity. The main absorption peak of PUEP at 276 nm is associated with the interaction of tyrosine, tryptophan, and phenylalanine (Dabbour, He, et al. 2020), corresponding to new shoulder peaks due to π‐π* energy level jumps attributed to fine structure vibrations. Both PUEP and TP show a sharp increase in absorbance below 250 nm, with terminal absorption in the far ultraviolet (Tarek et al. 2023).

#### 3.4.9 Microstructure of PUEP (SEM)

**FIGURE 5:**
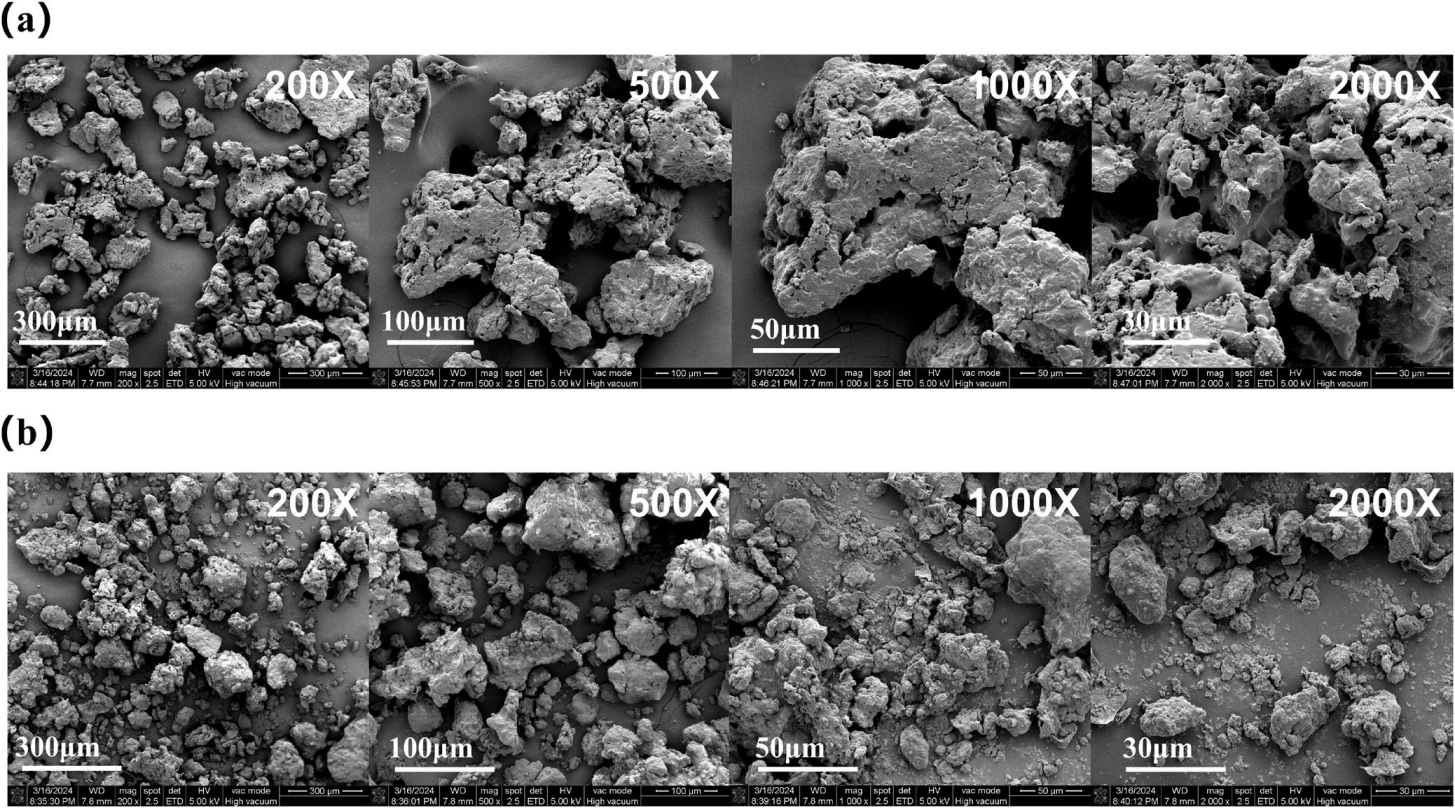
Protein microscopic morphology at different magnifications: (a) TP; (b) PUEP.

As shown in Figure 5, the microstructure of the *Pinus pumila* nut protein was analyzed at different magnifications using scanning electron microscopy (SEM). The overall apparent morphology of *Pinus pumila* nut protein is rough and granular, containing randomly distributed microporous structures that link protein molecules into larger molecular clusters with localized pores. The structure of TP is compact, with cross‐linked surfaces, a high degree of polymerization, no obvious gaps, and regular edges, which is consistent with its low solubility. PUEP were more loosely structured, which was attributed to the enzymatic treatment and ultrasonic cleavage of the inter‐protein cross‐linking structure, which disrupted the inter‐protein hydrogen and disulphide bonds, resulting in more disordered structures and irregular fragments. At the same time, the ultrasonic modification increased the irregularity of the edges of PUEP and the appearance of a cavity structure between the molecules, facilitating their use in the food industry, which is consistent with the results of ultrasonic‐assisted cellulase extraction of mulberry leaf proteins (Fan et al. 2023). The shape, structure density, and particle size of PUEP were significantly altered compared to TP, but the surface roughness did not change much.

### 3.5 Amino Acid Analysis

The amino acid content and composition of proteins is a direct reflection of the quality and balance of protein composition. As can be seen in Table 5, 17 amino acids have been successfully detected in the protein of *Pinus pumila* nuts. A full range of amino acids was identified. Tryptophan, asparagine, and glutamine were not detected due to hydrolytic destruction of the amide group by hydrolysis. There was no significant difference between the amino acid content of PUEP and TP. The EAA content of PUEP was 26.19 g/100 g, the NEAA content was 64.98 g/100 g, and the four amino acids with the highest content were: Glu > Arg > Asp > Leu. Glutamic acid, after being absorbed by the human body, is easily formed into glutamine with blood ammonia, which can alleviate the toxic effects of ammonia in the metabolic process, protect the liver, and can be used as an energy substance for brain tissue to improve the maintenance of brain function. Arginine can alleviate cellular inflammation and death, and not only participates in protein synthesis, but also promotes the development of animal immune organs, the proliferation and differentiation of T lymphocytes, and immunomodulatory functions. The amino acid composition indicates that the *Pinus pumila* nut protein has good biological activities such as neurostimulation and immunostimulation, which can be used to extract high quality active products.

**TABLE 5:** Content of Amino Acid of TP and PUEP.

	TP (g/100 g)	PUEP (g/100 g)
Thr	3.02 ± 0.04	2.46 ± 0.07
Val	3.56 ± 0.06	3.19 ± 0.07
Met	2.04 ± 0.10	1.98 ± 0.02
Ile	4.15 ± 0.07	3.46 ± 0.08
Leu	7.20 ± 0.09^d^	6.57 ± 0.07^d^
Phe	3.98 ± 0.06	3.67 ± 0.03
Lys	5.00 ± 0.05	2.49 ± 0.05
His	3.39 ± 0.04	2.37 ± 0.04
Asp	9.22 ± 0.14^c^	7.80 ± 0.09^c^
Ser	4.89 ± 0.06	5.06 ± 0.08
Glu	20.88 ± 0.32^a^	20.62 ± 0.30^a^
Gly	3.69 ± 0.06	3.76 ± 0.05
Ala	4.15 ± 0.08	3.59 ± 0.05
Cys	2.37 ± 0.49	1.83 ± 0.14
Tyr	3.47 ± 0.05	3.57 ± 0.04
Arg	13.94 ± 0.17^b^	14.85 ± 0.21^b^
Pro	3.96 ± 0.10	3.90 ± 0.06
Total amino acids	98.92	91.17

*Note:* The data in this table are analyzed by one‐way ANOVA test, and different letters in the same column indicate significant differences (*p* < 0.05).

In PUEP, the content of hydrophilic amino acids was 12.92 g/100 g, hydrophobic amino acids 30.12 g/100 g, basic amino acids 19.71 g/100 g, acidic amino acids 28.42 g/100 g, aromatic amino acids 7.24 g/100 g, branched chain amino acids 13.22 g/100 g. The abundance of hydrophobic and branched chain amino acids in *Pinus pumila* nut protein may enhance its interfacial action and biological activity. *Pinus pumila* nut proteins are rich in NEAA (e.g., glutamate, proline, arginine), but “NEAA” is not absolute, especially in subhealthy or critically ill patients; NEAA play an important role in the regulation of gene expression, cell signaling, antioxidant response, fertility, neurotransmission, and immunity. Glu and Arg account for 38.90% of the amino acid content of PUEP, both of which promote the production of NO, which can enhance the phagocytosis of leukocytes and have a profound biological effect on the microbial cycle. Glutamic acid is also indicated for the restorative treatment of patients with sepsis, organ system failure, and intestinal damage (Neu et al. 1996). The sweet amino acid content of PUEP was 41.88 g/100 g in the amino acid flavor analysis, which indicates that it can be used directly in the food industry without additive flavor modification.

## 4 Conclusion

*Pinus pumila* nut protein was extracted using the low‐power ultrasound‐assisted papain method, and the process‐optimized yield was almost three times higher than that of the conventional method, with a high extraction rate of 83.72%. The solid–liquid ratio was an important factor influencing the extraction rate of PUEP. The functional properties of PUEP, such as solubility, ESI and EAI, FC and FS, WHC and OHC, were significantly improved, which were related to the increase in surface hydrophobicity, the decrease in PDI value, the decrease in relative β‐sheet content, and the decrease in sulfhydryl group content. Basic research on the functional properties of PUEP can help to promote its practical application in the food industry, and its excellent functional properties in salt and acid–base solution will make PUEP a potential raw material for foam stabilizers, food gels, Pickering emulsions, edible films, and vegetarian meats, etc. However, it is still necessary to investigate further modifications of the extraction method or modification method to improve the functional properties of *Pinus pumila* nut protein in H_2_O.

## Author Contributions

**Zhe‐Xuan Mu:** writing – original draft (equal), writing – review and editing (equal). **Bing‐Xiao Liu:** project administration (equal). **Shuang‐Ding Lai:** project administration (equal). **Zhen‐Yu Wang:** supervision (equal). **Xiao‐Hong Lv:** supervision (equal). **Lin Yang:** funding acquisition (equal). **Hua Zhang:** resources (equal).

## Ethics Statement

The authors have nothing to report.

## Conflicts of Interest

The authors declare no conflicts of interest.

## Data Availability

The article contains all experimental data supporting the results of this study. Detailed study protocols and parameter descriptions are available from the corresponding author upon reasonable request.
